# Risk-based approach to school entry examinations in Germany – a validation study

**DOI:** 10.1186/s12887-019-1825-8

**Published:** 2019-11-19

**Authors:** Amand Führer, Andreas Wienke, Snezhina Wiermann, Christine Gröger, Daniel Tiller

**Affiliations:** 1 Martin-Luther-University Halle-Wittenberg, Institute of Medical Epidemiology, Biometrics and Informatics, Halle (Saale), Germany; 2 Public Health Department, City of Halle (Saale), Germany

## Abstract

**Background:**

In Germany, all preschoolers undergo a school entry examination (SEE). While most children are sufficiently served with standardized developmental tests only, for a small group of otherwise underserved children, the SEE should also include a subsidiary health checkup. The aim of the study was to validate selection criteria to differentiate these two groups of children.

**Methods:**

Secondary data from the SEEs of 2016 and 2017 that contained information on 3513 children were analyzed. Of these children, a subset was selected in which no severe developmental disorders were diagnosed prior to the SEE (*n* = 2744). The selection criteria identified in an earlier study (low or medium social status, missed the last pediatric routine check-up, migration background, three or more siblings, and raised by a single mother) were then applied to this subset to estimate their effectiveness in finding children at risk for a newly diagnosed severe developmental disorder. The sensitivity, specificity and positive and negative predictive values of the selection criteria were calculated.

**Results:**

The tested selection criteria identified children who would likely benefit from a subsidiary checkup in the context of SEEs with a sensitivity of 96% (95% CI: 94.5–98.9%). The negative predictive value and specificity of the criteria were 99% (98.6–99.7%) and 34% (32.1–35.8%), respectively. By using this approach, the number of children seen by a physician could be reduced to 53% of the age cohorts.

**Conclusion:**

The tested selection criteria are a viable way to differentiate children for whom SEEs should include a subsidiary health checkup from those who do not need it. Therefore, the time that physicians spend with SEEs could be reduced. Using the selection criteria to establish a stepped procedure in SEEs therefore offers a valid way to focus physicians’ resources on the children who need them most.

## Background

School entry examinations (SEEs) are obligatory examinations in Germany, where all children are examined by the respective communal departments of public health (*Gesundheitsamt*) before starting school. Similar examinations are also common in other countries, while the logistic specifics and the content of the examinations differ from place to place: California, for instance, employs an approach highly similar to that of Germany [1], while in other countries, SEEs are not obligatory or include a different set of procedures [2, 3].

Within Germany, the details of the examination also vary from federal state to federal state, while they all share a number of partly conflicting goals: First, SEEs are intended to identify developmental delays that could interfere with the child’s educational success [4–7]. This function of the SEE is sometimes referred to as the “*occupational health* function” [8]. Second, through the coverage of a whole birth cohort, valuable and otherwise only difficult-to-obtain *epidemiological* data are gathered [6, 9–11]. Third, by being an obligatory examination, SEEs are also intended to provide individual medical help to children who are otherwise underserved [12, 13] and thereby function as a *subsidiary pediatric checkup* [14].

These different functions of SEE are in conflict due to the interfering logistic and administrative necessities that they imply: While the occupational health function and the epidemiological function of SEEs are best served by a standardized procedure that covers all children (but does not necessarily require a physician), the subsidiary function would optimally target only a subgroup of underserved children who might need a physician’s attention. Nevertheless, at the moment, most federal states of Germany follow a one-size-fits-all approach, where all children undergo both standardized testing *and* a subsidiary checkup. This approach leads to the paradox situation in which a substantial part of the resources of public health departments is spent on examining children who are already under regular and thorough pediatric care [15], while children with health needs might not receive the support that would improve their situation because resources are not sufficient, e.g., for adequate follow-up. Additionally, communal departments of public health report a lack of resources to offer preventative services and health promotion programs [9, 16], which again would benefit especially disadvantaged children. Clearly, this situation raises questions about distributional justice [17, 18] and the role that departments of public health could play in reducing health inequities [19, 20].

In addition to these problems, critics argue that SEE are redundant [15] because they are in parallel to recommended but voluntary pediatric checkups that range from the first day of life (“U1”) to kindergarten age (up to “U9”). The similarities between SEE and U8 and U9 have been used as an argument against an obligatory SEE for children who underwent voluntary checkups [9]. Similar to the SEE, the U8 includes (at the age of four) a physical examination, hearing and vision tests, and developmental tests for motor function and language, while the U9 (in the sixth year of life) consists of a physical examination, a vision test and developmental tests for fine and gross motor skills, as well as cognitive and social development and different dimensions of language.

### Aim of the study

This situation appears to be inefficient and undesirable. Therefore, in an earlier study, we used data from the SEE in Halle (Saale), Germany, in 2015 to develop selection criteria that would identify high-risk children, and we outlined a risk-based approach to SEEs, where all children undergo standardized testing for developmental delays (whereby the needed epidemiological data are gathered), while only the children at increased risk receive a subsidiary pediatric checkup [21]. In a first analysis, these selection criteria proved to be 100% sensitive in identifying high-risk children, while the SEE-related workload of physicians in the communal public health department would be reduced to 37% of the workload using a blanket approach.

The aim of the current work is to validate this approach using the data of the SEEs of the two subsequent years, 2016 and 2017.

## Methods

### Preliminary considerations

Central to any risk-based approach must be a definition of what constitutes the respective risk. In our case, the question centers around the following question: Which children would benefit from a subsidiary pediatric checkup, and which would not?

Clearly, children who already are in proper pediatric care and whose parents, nursery school teachers or other educators follow the child’s development and health attentively do not need yet another checkup by a physician who knows little about the child’s history and social background. Meanwhile, for children whose parents do not take them to routine checkups and whose environment might be inattentive to health or developmental problems, a subsidiary checkup might be an important event for setting the course of their future health.

Therefore, we followed Rosenkötter et al.’s [22] suggestion and assumed that the existence of already diagnosed developmental delays can be seen as a surrogate parameter for functioning access to health care services. By implication, we assumed that the diagnosis of a heretofore unknown severe developmental delay in the SEE is an indicator of insufficient preventative health care and an indication for the need for a subsidiary checkup.

This approach might slightly overestimate the lack of preventative health care for those few children who are designated to receive their U9 after the SEE.

### Data

In 2016 and 2017, the communal Public Health Department of Halle (Saale), Germany, examined a number of 2013 and 2006 preschoolers. Of those children, parents refused the use of the data for secondary analysis in 272 and 234 cases. Therefore, the pooled dataset of both years included data on 3513 children. Data contained information gathered from a parental questionnaire, a standardized examination including vision, hearing, and developmental tests, and a clinical examination.

Children with known preexisting developmental disorders (*n* = 769) were excluded from the dataset to derive a dataset containing only children in whom a *heretofore unknown* severe developmental disorder (for definition see below) could have been diagnosed in the SEE.

The final dataset therefore included information on 2744 children.

### Variables

The procedure for selecting variables potentially associated with increased risks for severe developmental disorders was developed in an earlier work [21], and variables are described in greater detail there. For three composed secondary variables, we employed the definitions as they are routinely used by the department of public health and the federal health reporting system:

Social status according to the “Brandenburger Sozialindex” [23] was established using the information on parents’ level of education and employment status. This rather broad estimate of a child’s socioeconomic situation was chosen, since it is routinely used within the SEE in Saxony-Anhalt to classify children’s social status and is also used in many other federal states, even though this approach has been criticized by some authors for being too simplistic [24, 25]. Since low and medium social status were both found to be associated with an elevated risk for developmental delays, they were grouped together and contrasted to high social status in the following analysis.

As information on the children’s native language or the language spoken at home are not routinely collected and were therefore not available for our analysis, migration background was classified using the information on each parent’s nationality and the parents’ and the child’s country of birth following the standard procedure prescribed by the German Federal Statistical Office [26].

Hereby we use the definition of migration background as it is routinely used within SEE in Saxony-Anhalt, even though this definition has been criticized for being too broad [27, 28]. A more detailed analysis of – and recommendations for – how to define migration status with the information routinely available from SEE data is currently submitted elsewhere (Führer A, Tiller D, Brzoska P, Korn M, Gröger C, Wienke A (2019) Health-related disparities among migrant children at school entry in Germany. How does the definition of migration status matter? forthcoming).

The most important clinical outcome variable, “severe developmental disorder”, was defined following the routine procedure used in the SEE in Saxony-Anhalt, where such a disorder is defined as a combined endpoint built on the standardized tests for gross and fine motor skills, cognitive functions, diction and grammar [29]. According to this definition, children classified as suffering from a severe developmental disorder are developmentally delayed in more than one of the dimensions and require complex interventions.

### Statistical analyses

To validate the selection criteria identified in an earlier study [21], the proposed procedure was administered to this new dataset in three steps: First, high-risk children were identified using the criteria under validation. Each child who fulfilled at least one of the following criteria was considered high-risk: low or medium social status, missed the last pediatric routine check-up, migration background, three or more siblings, and raised by a single mother [21].

Second, the prevalence of severe developmental disorders was calculated. Third, the selection criteria’s sensitivity, specificity, positive and negative predictive values for a newly diagnosed severe developmental disorder were derived using a two-times-two table, and the number of children seen by a physician in a risk-based, stepped SEE was calculated. Confidence intervals are calculated using the Wilson score method [30].

Analyses were performed using SAS® (Cary, North Carolina, USA).

## Results

Demographic characteristics of the whole cohort and its subpopulations of children with and without already established severe developmental disorders are shown in Table 1. As the table shows, sociodemographic characteristics of the cohort *without* preexisting developmental findings and the cohort *with* preexisting developmental findings differ in virtually all displayed variables. Among children with preexisting findings, there are proportionally more boys, fewer children classified as high social status, fewer children with a migration background, more children with more than three siblings, and fewer children living with both parents.

**Table 1:** Sociodemographic characteristics of the age cohort

	Children without heretofore known severe developmental disorders *n* = 2744		Children with heretofore known severe developmental disorders = 769		Whole age group *n* = 3513	
	Number	Percent	Number	Percent	Number	Percent
Sex						
Male	1334	48.6	473	61.5	1807	51.4
Female	1410	51.4	296	38.5	1706	48.6
Age (years)						
4	211	7.7	46	6	257	7.3
5	2318	84.5	680	88.4	2998	85.3
6	212	7.7	42	5.5	254	7.2
7	0	0	1	0.1	1	0.03
Social status (Brandenburger Sozialindex)						
High	1194	48	234	33.4	1428	44.7
Medium	841	33.8	252	36	1093	34.3
Low	455	18.3	215	30.7	670	21
Migration background	519	18.9	93	12.1	612	17.4
Number of Siblings						
< 3	2374	86.5	622	80.9	2996	85.3
> =3	370	13.5	147	19.1	517	14.7
Parents‘marital status						
Both parents	1934	71.4	467	61.1	2401	69.2
Single mother	564	20.8	216	28.2	780	22.5
Single father	41	1.5	15	2	56	1.6
Other^a^	168	6.1	67	8.7	235	6.7
Weight according to [29]						
Underweight	288	10.7	87	11.7	375	10.9
Normal weight	2192	81.1	570	76.4	2762	80.1
Overweight	130	4.8	47	6.3	177	5.1
Adiposity	94	3.5	42	5.6	136	3.9
Mean (kg):	20.4	s.d.: 3.5	20,69	s.d.: 3.9	20,45	s.d.: 3.6
Height (cm)	114.7	s.d.: 5.4	114,35	s.d.: 5.5	114,6	s.d.: 5.4
Participation in routine checkup 8	2036	85.7	653	88.6	2689	86.4
Participation in routine checkup 9	1564	65.8	528	71.6	2092	67.2

^a^‘other’ includes mother with new partner, father with new partner or other caregivers

**Fig. 1:**
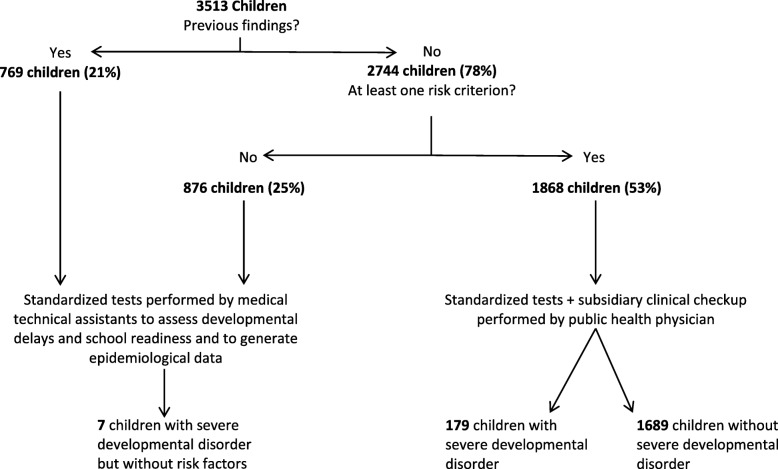
Application of the selection process on data from 2016 and 2017

Using the selection process suggested in [21], Fig. 1 displays the pathways that preschoolers of 2016 and 2017 would have taken in the school entry examination and their respective numbers. It is shown that by using the suggested selection criteria, only 53% (*n* = 1868) of the cohorts of these 2 years’ preschoolers – instead of all children – would have to be seen by a physician, while 47% of all children (*n* = 1645) would undergo only standardized testing by a medical technical assistant.

Further analysis shows that this procedure not only reduces the number of children subject to clinical examination but also has high sensitivity to select high-risk children: Of 186 children in the cohort who suffer from a heretofore unknown severe developmental disorder, 179 would have been identified correctly by the selection criteria, resulting in a sensitivity of 96%. In addition, Table 2 shows the two-times-two table and a specificity of 34%, a positive predictive value of 9.6% and a negative predictive value of 99%.

**Table 2:** Sensitivity and specificity of the selection criteria

	At least one risk factor		
Heretofore unknown severe developmental disorders	yes	no	total
yes	179	7	186
no	1689	869	2558
total	1868	876	2744
	Sensitivity:	0.962 (95% CI: 0.935–0.989)	
	Specificity:	0.339 (95% CI: 0.321–0.358)	
	PPV:	0.096 (95% CI: 0.082–0.109)	
	NPV:	0.992 (95% CI: 0.986–0.997)	

## Discussion

The aim of this paper was to validate the selection criteria identified in an earlier work. By showing that the application of the selection criteria results in a high sensitivity of 96% in the 2 years analyzed, we conclude that the suggested risk-based approach to school entry examinations is a viable way of organizing SEE as a stepped procedure that reduces physicians’ time spent on school entry examination without leaving high-risk children behind.

While the positive predictive value and the specificity of the proposed selection criteria are relatively low, we tend to consider this issue to be a matter of minor importance: Considering that to date, all children routinely undergo the SEE and that it does not involve any invasive or painful diagnostics, being classified false positive does not cause any harm. In contrast, we emphasize that compared to the current routine in many German departments of public health, our proposed selection procedure would considerably reduce the time physicians spend examining low-risk children, while almost all children with developmental delays would be identified.

Similar conclusions have been drawn from SEE data in the UK more than two decades ago [31], where employing physicians to screen all children has been found to be inefficient to detect currently unknown health conditions [32–34], while the implementation of a selective approach reduced the number of examined children to between 20 and 40% of the age cohort [35]. Subsequently, school doctors’ ability to focus their time and resources on high-risk children was strongly increased [31].

These discussions also resonated within the German public health sector and led to some federal states’ introduction of selective procedures for SEEs. Our approach differs from those methods by using *five* risk factors to define high-risk children: Other federal states of Germany only use a child’s participation in the last routine checkup to decide about his or her being seen by the physician [36]. While these approaches achieve even larger reductions in physicians’ workload compared to our risk-based approach, using checkup participation as the only criterion would result in a sensitivity of less than 65% in our preschooler population. Consequently, 66 out of 186 high-risk children would not have been seen by a physician in the school entry examination.

On the other hand, evaluations of already established approaches to SEE that also employ a stepped procedure reach similar conclusions and show that stepped procedures that take the children’s risk as a starting point can indeed serve to “employ physicians’ workforce in a problem-centered way” [11]: In the federal state of Baden-Wuerttemberg, for instance, only children who display abnormalities in a set of standardized tests performed by a medical technical assistant are seen by a physician in a last step. In this instance, the sensitivity is 95% [11, 37].

Establishing a stepped procedure for SEE would therefore improve SEE’s function as subsidiary pediatric checkups. Their epidemiological function on the other hand might be slightly impaired by the restriction of data collection to the standardized tests. Since the epidemiological usefulness of data derived from physical examinations in SEE has been questioned due to a lack of reliability [10, 38], we consider this to be a minor limitation.

At this point, it seems important to us to highlight that while emphasizing the necessity to focus physicians’ resources, our argument is not driven by economic considerations. Instead, our argument is built on the belief that the public health sector should orient its work structures around the idea of health equity which, according to Whitehead, demands “resource allocation in relation to social and health needs” [39]. In line with the assumption that health equity ultimately means to eliminate “systematic disparities in health” [40], we suggest that the one fits all-approach to SEE poses ethical questions that can best be answered by analyzing existing health disparities and adjusting work structures to ensure that resources are made available to disadvantaged children [41]. As our validation shows, the proposed risk-based approach to SEEs can be a step in this direction.

## Conclusion

Considering that scarce resources, especially the lack of medical personnel, are a problem in the public health sector, we want to highlight that our risk-based approach can reallocate resources without endangering the sensitivity of the school entry examination and thus enable public health physicians to focus the scope of their activities on the subsidiary dimension of their work.

## Data Availability

The data that support the findings of this study are available from the Public Health Department, City of Halle (Saale), Germany (address: Fachbereich Gesundheit, Niemeyerstraße 1, 06110 Halle (Saale), Germany), but restrictions apply to the availability of these data, which were used under license for the current study and therefore are not publicly available. Data are, however, available from the authors upon reasonable request and with permission of the Public Health Department, City of Halle (Saale), Germany.
